# Towards a decision support system for post bariatric hypoglycaemia: development of forecasting algorithms in unrestricted daily-life conditions

**DOI:** 10.1186/s12911-025-02856-5

**Published:** 2025-01-20

**Authors:** Francesco Prendin, Olivia Streicher, Giacomo Cappon, Eva Rolfes, David Herzig, Lia Bally, Andrea Facchinetti

**Affiliations:** 1 Department of Information Engineering (DEI), University of Padova, Via G. Gradenigo 6/B, Padua, 35131 Italy; 2 Department of Diabetes, Endocrinology, Nutritional Medicine and Metabolism, Inselspital, Bern University Hospital, University of Bern, Bern, Switzerland

**Keywords:** Post bariatric hypoglycaemia, Data-driven forecasting models, Continuous glucose monitoring

## Abstract

**Background:**

Post bariatric hypoglycaemic (PBH) is a late complication of weight loss surgery, characterised by critically low blood glucose levels following meal-induced glycaemic excursions. The disabling consequences of PBH underline the need for the development of a decision support system (DSS) that can warn individuals about upcoming PBH events, thus enabling preventive actions to avoid impending episodes. In view of this, we developed various algorithms based on linear and deep learning models to forecast PBH episodes in the short-term.

**Methods:**

We leveraged a dataset obtained from 50 patients with PBH after Roux-en-Y gastric bypass, monitored for up to 50 days under unrestricted real-life conditions. Algorithms’ performance was assessed by measuring Precision, Recall, F1-score, False-alarms-per-day and Time Gain (TG).

**Results:**

The run-to-run forecasting algorithm based on recursive autoregressive model (rAR) outperformed the other techniques, achieving Precision of 64.38%, Recall of 84.43%, F1-score of 73.06%, a median TG of 10 min and 1 false alarm every 6 days. More complex deep learning models demonstrated similar median TG but inferior forecasting capabilities with F1-score ranging from 54.88% to 64.10%.

**Conclusions:**

Real-time forecasting of PBH events using CGM data as a single input imposes high demands on various types of prediction algorithms, with CGM data noise and rapid postprandial glucose dynamics representing the key challenges. In this study, the run-to-run rAR yielded most satisfactory results with accurate PBH event predictive capacity and few false alarms, thereby indicating potential for the development of DSS for people with PBH.

**Supplementary Information:**

The online version contains supplementary material available at 10.1186/s12911-025-02856-5.

## Background

Bariatric surgery is a potent anti-obesity treatment resulting in durable weight loss and improvement, or even resolution, of obesity-related comorbidities such as type 2 diabetes [1]. Despite these benefits, the surgery-related anatomical rearrangements induce substantial glycaemic variability in response to food intake [2]. In a subset of patients, this high postprandial glycaemic variability is linked with episodes of hypoglycaemia, typically occurring 90–150 min after carbohydrate intake [3]. The condition, also known as postbariatric hypoglycaemia (PBH), is more common in people undergoing Roux-en-Y gastric bypass (RYGB) but can also occur after sleeve gastrectomy [4]. Episodes of PBH are characterized by rapid, meal-induced rises in blood glucose (BG), followed by a sharp decrease to hypoglycaemic levels (i.e., BG < 54 mg/dL). Hypoglycaemia can predispose to acute health hazards (e.g. accidents, falls) and, if recurrent, may be also a risk factor for neurocognitive decline and cardiovascular comorbidity [5]. Although most evidence indicating hypoglycaemia-related comorbidity stems from studies in people with diabetes, PBH was recently shown to impair driving performance when compared to stable normoglycaemia, in a vehicle simulation environment [6]. Additionally, recurrent episodes of PBH can lead to anxiety, dependencies and constraints in daily activities with negative impact on quality of life [6, 7]. Thus, forecasting PBH events by means of glucose prediction algorithm in daily life may provide helpful assistance by alerting people to take preventive or corrective actions. Glucose prediction algorithms are usually fed by continuous glucose monitoring (CGM) data, which can trace glucose trajectories with high resolution (e.g. every 5min) [8]. Unlike for people with diabetes, [9, 10] the development of hypoglycaemia predictive algorithms for the PBH population is at its early stage, with only two main literature contributions. The first one is a heuristic-based algorithm directing glucagon in response to predicted or detected low glucose levels [11]. The other one is a proof-of-concept study from our research group which demonstrated the feasibility of developing PBH predictive algorithms based on black-box models using noise-free CGM data in a small sample size [12]. Moving forwards towards real-world application, the aim of this work was to develop a CGM-based algorithm for real-time prediction of PBH events in a larger, unfiltered dataset generated by 50 individuals under daily life conditions. To this end, we explored various forecasting algorithms based on linear and deep learning models and provided a robust evaluation of their performance.

## Methods

### Dataset

The data was collected within a prospective observational clinical study (NCT05212207) involving 50 post-RYGB adults with a confirmed diagnosis of PBH. Participants wore the Dexcom G6 CGM sensor (Dexcom Inc., San Diego, CA, USA) for up to 50 days, which was linked to IMPACT [13], a dedicated mobile application integrating data from CGM with input from other connected devices (e.g., smartwatches) and manually logged events (e.g., meal intakes, symptoms, etc.). During the data collection period, participants were blinded to their CGM values and were asked to follow their usual habits without study-specific modifications. None of the participants were on pharmacotherapies that interfered with glucose metabolism. We only included individuals with CGM data availability of more than 10 days to avoid imbalanced data contributions, leading to exclusion of data from 3 participants. Table 1 summarizes the key glucometrics of the included participants (*n* = 47).

**Table 1:** Dataset characteristics. Metrics are reported as median [25th-75th percentiles] for *n* = 47 PBH study participants. Abbreviations: standard deviation (std), mean amplitude of glucose excursion (MAGE), Level 1 hypoglycaemia: sensor glucose < 70mg/dL, Level 2 hypoglycaemia: sensor glucose < 54mg/dL

**CGM availability (days)**	39.5 [31.0–45.0]
**Mean Glucose [mg/dL]**	106.5 [103.1–116.8]
**Std Glucose [mg/dL]**	32. 5 [28.2–38.6]
**MAGE [mg/dL]**	55.3 [40.5–78.2]
**Time in Level 1 Hypoglycaemia (%)**	4.3 [2.7–7.6]
**Time in Level 2 Hypoglycaemia (%)**	1.0 [0.5–1.4]
**Time with glucose levels 70–180 mg/dL (%)**	90.1 [87.1–93.1]
**Time with glucose levels > 180mg/dL (%)**	4.2 [2.5–7.4]
**Total number of Level 2 Hypoglycaemic episodes**	821
**Duration of Level 2 Hypoglycaemic episodes (min)**	25 [20–30]

As shown in Table 1, median duration of CGM availability was 39 days. Mean glucose was 107 mg/dL, in line with levels of non-operated healthy individuals [14], but the std of the CGM traces (32.5 mg/dL) and the elevated MAGE (55 mg/dL), which measures the major BG fluctuations from peak to nadir, indicated a high glucose variability in the PBH population. The total number of Level 2 hypoglycaemic episodes amounted to 821 (i.e., almost 4 every 10 days per subject), with a median duration of 25 min.

For the evaluation of the algorithms’ performance, the data of each eligible subject was split into a training (70%, i.e., 28 monitoring days on average), a validation (15%, i.e., 5 monitoring days on average), and a test set (15%, i.e., 5 monitoring days on average). This resulted in a total of 520 PBH episodes included in the training, 134 in the validation and 167 in the test set.

During the monitoring period, common real-life challenges such as data gaps from transmission failures or CGM device replacements, and irregular sampling due to temporary sensor issue, may occur. For this reason, original CGM data were aligned to a 5-min time grid. Then, on the training set, data gaps shorter than 30 min were interpolated with a first-order polynomial, while for the test set, a causal zero-order-hold extrapolation was used.

### Glucose prediction models

Based on our previous work [12], we implemented and tested the following models for the real-time prediction of future glycaemia and the generation of hypoglycaemic alerts: a run-to-run approach based on an autoregressive model with recursive parameter estimation (rAR) [15], which is an adaptive method based on a well-known recursive scheme [16]; an autoregressive integrated moving average (ARIMA) model [17], which was the best performer in our previous work [12]; and a feed forward neural network (NN) [18]. In this study, we also investigated the use of two deep learning models: a long-short term memory (LSTM) neural network and a convolutional neural network stacked to a LSTM (CNN-LSTM). These methodologies have proved to be effective in predicting glucose levels and hypoglycaemic events in people with type 1 diabetes due to their ability to learn both short and long-term dependencies in time-series data [19–22]. For these reasons, once tailored to PBH individuals, deep learning algorithms should enable a more in-depth description of the nonlinear dynamics of the glucose-insulin systems [23, 24] as well as the rapid glycaemic excursions that characterize PBH episodes after meal ingestion. As an additional contribution, Random Forest (RF) and Light Gradient-Boosting Machine (LGB), representing bagging and boosting models respectively, were included in the comparison.

In the following, a detailed description of the final structure and hyperparameters of the predictive models developed in this work has been reported.

- **rAR:** rAR model employs a first-order autoregressive model based on recursive parameter estimation. A key element of rAR is the forgetting factor $\mu$, which is typically used to model nonstationary signals and it regulates the “memory” of the system. Specifically, the forgetting factor is in the range (0, 1) and it exponentially discounts old measurements such that a CGM sample that is $\tau$ samples old has a weight of ${\mu }^{\tau }$. Hence, CGM measurements older than $\tau = \frac{1}{1-\mu }$ have only a minimal influence to model parameter estimation and model prediction. For the current study, after a grid search approach (grid ranges [0.1–0.9]), we selected $\mu$ = 0.675, thus indicating that the 3 most recent CGM samples have a large impact for parameter estimation and prediction.
- **ARIMA**: the order of the ARIMA model were selected by minimizing the Bayesian Information Criterion using an exhaustive grid-search approach. In particular, the following ranges for the AR (p), I (d) and MA (q) part were considered: *p* = [1–15], q = [0–1], d = [1–15]. The final structure is an ARIMA model of *p* = 3, d = 1, q = 1. It is interesting to see that the order of the AR part of the ARIMA model indicates that the last 3 CGM samples contribute to the model prediction, similarly to the rAR.

For NN, LSTM and CNN-LSTM models, manual hyperparameter tuning was performed based on our expertise on glucose forecasting in type 1 diabetes. More than 20 different hyperparameter sets were tested, varying number of layers, neurons per layers, optimizers, learning rates, and batch sizes. The output of the optimization step yields to the following structures:

- **NN**: two hidden layers of 32 and 16 neurons with exponential linear unit (ELU) and linear activation functions for the first and the second layer, respectively;
- **LSTM**: two hidden layers of 24 and 12 neurons with ELU and standard activation functions for the first and the second layer, respectively;
- **CNN-LSTM**: two blocks of stacked convolutional layers (equipped with 16 and 8 filters), batch normalization (to center and rescale the input for reducing the internal covariance shift) and a pooling layer followed by a set of LSTM layers of 12 and 6 neurons with ELU activation functions.

For all these models, the output is a fully connected layer with 6 neurons (i.e., 30-min prediction horizon). The training is carried out for 300 epochs with an early stop point by monitoring changes in validation loss throughout a 30-epoch period. A batch size of 32 is used to optimize parameters using Root Means Squared Propagation with and initial learning rate of 1e-5.

The NN, LSTM and CNN-LSTM, have been developed within Python (Keras library) and trained using a Root Mean Squared Propagation (RMSprop) optimization algorithm on a Nvidia Titan RTX.

Concerning RF and LGB, more than 15 different sets of hyperparameters were evaluated. Particularly, for RF, these included number and max depth of tree, minimum number of observations in any node and minimum number of samples in the leaf node. The sets of hyperparameters for LGB included the number of boosting trees, learning rate, number of leaves for each tree. Additionally, we opted for leaving the trees growing until they reach the maximum number of leaves. The identification of the best set of hyperparameters led to the final structures:

- **RF**: the RF model is composed by 100 trees, each of them limited to 15 levels and with at least 10 samples per split;
- **LGB**: the LGB model is composed by 200 trees, each of them with 31 leaves (the maximum depth for each tree) and a learning rate = 0.02.

RF and LGB were implemented in Python using sci-kit learn library and the lightGBM package, respectively.

As a final remark, the real-time application of all the proposed algorithms requires that all the pre-processing technique applied prior to the forecasting step be causal. Therefore, the anti-causal zero-phase filter employed in [12], cannot be practically implemented. In addition, any casual real-time filter would introduce a phase shift, which translates into a delay in the filtered CGM data with respect to the original one, limiting the practical and clinical utility of the predictive algorithms. Considering these points, we opted to use the original CGM data as the only input for the predictive algorithms.

### Hypoglycaemia forecasting pipeline

**Fig. 1:**
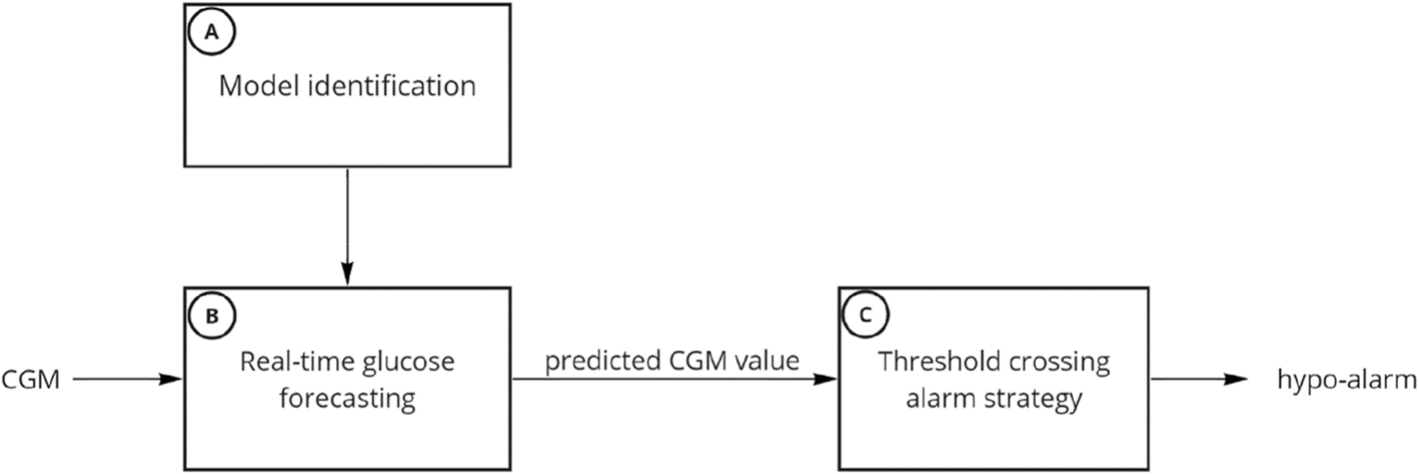
Pipeline for hypoglycaemia forecasting. Once the model has been identified on the training set (step A), it was used (step B) to forecast glucose at a certain prediction horizon (PH). Finally (step C), if the predicted concentration was below a suitable triggering threshold (AlarmLevel), the algorithm raises a hypo-alarm

The block scheme overview for hypoglycaemia forecasting is shown in Fig. 1 and consisted of three different phases. The first step (phase A) was the identification of a population-wise model (i.e., one model for each prediction methodology: rAR, ARIMA, NN, LSTM, CNN-LSTM, RF and LGB) using the training set. In accordance with the recently published consensus guidelines for artificial intelligence and machine learning developers in the field of diabetes [25], all the models were trained using only past CGM data to predict future sensor glucose levels. Particularly, linear models (rAR and ARIMA) have been identified by resorting to the state-of-art prediction error method (PEM) which requires the minimization of the one-step ahead prediction error. The deep learning models have been designed as sequence-to-sequence (seq2seq) models aimed to predict future glucose trajectory. This is a well-known strategy to deal with temporal sequences in deep learning for time series forecasting purposes [26] and it has been widely applied for glucose prediction in type 1 diabetes [27, 28].

The second step (phase B) was the prediction of future glucose levels at a specific prediction horizon (PH, i.e., how far ahead in time the algorithm forecasts), by simulating the acquisition of CGM data in real-time. While the identified linear models can forecast glucose levels by iterating the one-step ahead prediction up to the considered PH, the trained deep learning models can directly output the forecast sequence of future glucose sensor levels up to a maximum PH (i.e., 30 min in this work). Finally, the last step (phase C) was the generation of a hypoglycaemic alert when the forecasted glucose concentration was below a triggering threshold, named AlarmLevel. The AlarmLevel is a predictive threshold and serves as a hyperparameter within the glucose forecasting model, and its value can be set lower or higher than the hypoglycaemia threshold. When the predicted glucose level is forecasted to fall below this level, an alarm is triggered. The AlarmLevel should not be confused with the hypoglycaemia threshold, which serves as the clinical marker of hypoglycaemia, which was set to 54 mg/dL in the present study. Please note that the use of the validation and test set is intended only for phases B-C.

While receiving preventive alerts is crucial to avoid/mitigate the upcoming hypoglycaemia, getting too many alarms cause burden and frustration. To avoid multiple consecutive alarms, a shut-off mechanism, ignoring new alarms in the event of a recent one (i.e., within the timeframe of the PH) or if the current CGM value remained below the hypoglycaemic threshold, was implemented.

Differently from previously published work on PBH forecasting [12], here one of the novelties is that we consider both the PH and AlarmLevel as two hyperparameters of the hypoglycaemic predictive algorithms. In fact, as already demonstrated in studies about CGM data forecasting in people with diabetes [29], the fine tuning of hyperparameters (PH and AlarmLevel in this work) allows to increase the performance of the algorithms and compensate for inaccurately predicted glucose concentration (i.e., the output of phase B) that are common when dealing with data acquired in unrestricted daily-life conditions. In line with standard machine learning approaches, the validation set was used to learn the best combination of PH and AlarmLevel for each model. The tuning of PH and AlarmLevel is discussed in Sect. " Hyperparameters tuning: PH and alarmLevel".

Regression-based approaches for predicting hypoglycaemia are not new in the field. They have already been adopted in Laguna Sanz et al. [11] for the prediction/detection of PBH episodes and in our previous proof-of-concept study [12]. This framework is widely established in the field of type 1 diabetes [30–33] and is currently implemented in several commercial CGM systems [34]. Additionally, a recent study has shown that no clear advantages seems to emerge when using a standard binary classification framework instead of a regression-based one [35].

### Performance assessment criteria

An alarm was considered as a true positive (TP) if a PBH event occurred within 45 min after the raised alarm; and a false positive (FP) if no PBH events occurred within that time window. A false negative (FN) was defined as lack of alarm activation despite the occurrence of a PBH event. The algorithms’ performance was evaluated with the following metrics: i) Precision (describing the fraction of alarms correctly raised by the algorithm over the total number of raised alarms); ii) Recall (also known as sensitivity or true positive ratio, being the percentage of events correctly predicted over the total number of PBH events); iii) F1-score (the harmonic mean of the Precision and Recall); iv) False Alarms per day (FP/day) and v) Time Gain (TG, defined as the temporal distance between a TP alarm and the onset of a PBH event). The TG is particularly relevant as it represents the time window during which a patient can act to prevent an impending event.

Although the primary aim of this work is predicting PBH events, it could be useful to quantify the accuracy of the predicted profiles generated by the glucose predictive models described in Sect. " Glucose prediction models" (step B, Fig. 1). To this end, for each PH, we computed the mean absolute error (MAE) defined as:

$$MAE\left(PH\right)= \frac{1}{N}\left|\sum_{t=1}^{N}y\left(t+PH\right)-\widehat{y}(t+PH|t)\right|$$

where N is the length of the CGM trace, $y\left(t+PH\right)$ is the future glucose concentration and $\widehat{y}\left(t+PH|t\right)$ is the PH-step ahead prediction that is computed by using all the past glucose data up to the current time $t$.

### Hyperparameters tuning: PH and alarmLevel

A perfect glucose forecasting model would obviate the need to fine-tune PH and AlarmLevel hyperparameters because the predicted glucose levels would perfectly match with the future CGM values, resulting in a forecasting error of 0. The trivial consequence would be equality of TG and PH as well as AlarmLevel and hypoglycaemic threshold. However, a model identified on real-world data cannot be perfect by definition and, as described in Sect. " Glucose prediction models", highlighting the need for tuning of PH and AlarmLevel to ensure satisfactory PBH prediction performance. To achieve this goal, a grid-search approach has been implemented with PH ranging from 15 to 30 min and AlarmLevel spanning from 35 mg/dL to 80 mg/dL. For each pair (PH, AlarmLevel) and for each sensor glucose predictive model, the hypoglycaemic prediction performance was assessed on the validation set. Finally, the optimal combination of hyperparameters was determined as the one maximizing the F1-score on the validation set.

## Results

Tables 2 and 3 summarize the performance of the evaluated models on the validation and the test set, respectively.

**Table 2:** Performance for the best set of hyperparameters (PH, AlarmLevel) on validation set for the algorithms under investigation (rAR, ARIMA, NN, LSTM and CNN-LSTM). Results of TG are reported as median [25th-75th] percentile. Abbreviations: FP/day, false positives per day; TG, time gain; rAR, recursive Autoregressive model; ARIMA, Autoregressive Integrated Moving Average; NN, Neural Network; LSTM, Long Short-Term Memory Neural Network; CNN-LSTM, Convolutional Long Short-Term Memory Neural Network; RF, Random Forest; LGB, LightGBM

	**Performance Metrics**					**Best Hyperparameters**	
Model	**Precision (%)**	**Recall (%)**	**F1-score (%)**	**FP/day**	**TG (min)**	**PH (min)**	**AlarmLevel (mg/dL)**
rAR	68.24	86.57	76.32	0.2	10 [5–10]	25	42
ARIMA	60.38	71.64	65.53	0.3	10 [10–15]	15	52
NN	74.51	56.72	64.41	0.1	10 [10–15]	25	59
LSTM	82.69	64.18	72.27	0.1	10 [5–10]	15	57
CNN-LSTM	79.80	58.96	67.81	0.1	10 [5–10]	15	56
RF	79.13	67.91	73.09	0.1	10 [10–15]	15	58
LGB	79.83	70.89	75.09	0.1	10 [5–10]	15	56

**Table 3:** Performance on the test set with the best set of hyperparameters identified on the validation set. Results of TG are reported as median [25th-75th] percentile. Abbreviations: FP/day, false positives per day; TG, time gain; rAR, recursive Autoregressive model; ARIMA, Autoregressive Integrated Moving Average; NN, Neural Network; LSTM, Long Short-Term Memory Neural Network; CNN-LSTM, Convolutional Long Short-Term Memory Neural Network; RF, Random Forest; LGB, LightGBM

Model	Precision (%)	Recall (%)	F1-Score (%)	FP/day	TG (min)
rAR	64.38	84.43	73.06	0.17	10 [5–15]
ARIMA	44.87	70.66	54.88	0.31	10 [10–15]
NN	64.14	55.69	59.62	0.11	10 [10–15]
LSTM	68.97	59.88	64.10	0.1	10 [10–15]
CNN-LSTM	68.70	53.89	60.40	0.1	10 [5–10]
RF	70.07	57.49	63.16	0.08	10 [5–15]
LGB	67.52	63.47	65.43	0.1	10 [5–10]

### Hyperparameter assessment on the validation set

**Fig. 2:**
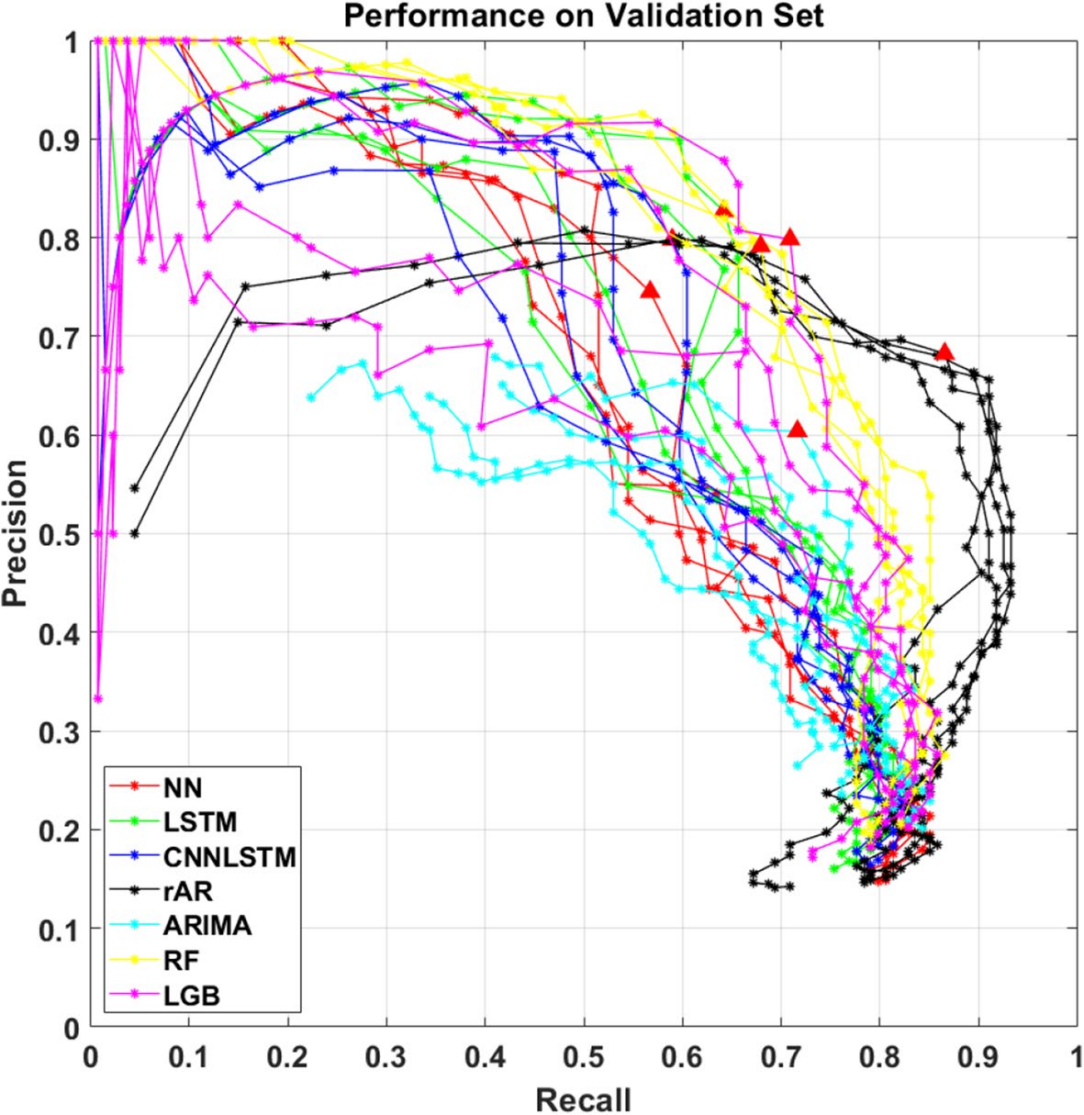
Tuning of PH and AlarmLevel on validation set. Precision-Recall plot for NN (red line), LSTM (green line), CNN-LSTM (blue line), rAR (black line), ARIMA (cyano line), RF (yellow line) and LGB (magenta line). The red triangle indicates the combination of hyperparameters that maximizes the F1-score

Figure 2 displays Precision and Recall for all tested models and combinations of hyperparameter values. As a common pattern, a rise of the AlarmLevel from 40 mg/dL to 80 mg/dL, let the models’ performance change from high Precision and low Recall to the opposite situation (i.e., high Recall and low Precision). In contrast, increasing PH did not have a clear impact on the model’s performance. Table 2 shows that rAR with the combination of PH = 25 min and AlarmLevel = 42 mg/dL achieved the best predictive performance among all the considered methodologies, with Precision = 68.24%, Recall = 86.57% and F1-score = 76.32%, a very limited number of FP/day (0.2) and a median TG of 10 min. Compared to rAR, ARIMA yielded an inferior Precision (about 60%) and Recall (about 72%), which combines resulted into a F1-score of 65.53%. Both the FP/day and the median time gain were in line with those of rAR (0.3 and 10 min, respectively). In this case, the best combination of hyperparameters was PH = 15 min and AlarmLevel = 52 mg/dL.

NN provided a F1-score = 64.41%, comparable to that of ARIMA, with higher Precision (74.51% vs 60.38%) and lower Recall (56.72% vs 71.64%) and a median TG of 10 min. Also, the number of FP/day (0.1) was slightly lower than both rAR and ARIMA.

LSTM achieved inferior performance than rAR in terms of Recall and F1-score (64.18% and 72.27%, respectively) but it was the best performing among the nonlinear models with Precision = 82.69% and TG = 10 min. Additionally, by granting the lowest number of FP/day in the validation set (i.e., 0.1), LSTM appeared to be a promising approach to predict PBH events. Finally, CNN-LSTM provided a larger Precision with respect to NN (79.80% vs 74.51%, respectively) and similar Recall (58.96% vs 56.72%, respectively). The number of FP/day was 0.1 (similar to LSTM) and the median TG = 10 min, in line with the other methodologies.

Compared to rAR and ARIMA models, RF provides a larger Precision (79.13% vs 68.24% vs 60.38%) that is in line with the Precision achieved by the other nonlinear methodologies which span from 74.51% (for NN) to 82.69% (for LSTM). In terms of Recall, RF achieved 67.91% which is larger than NN, LSTM and CNN-LSTM but it is inferior to the Recall provided by both rAR and ARIMA (86.57% and 71.64%, respectively). LGB demonstrated performance comparable to RF and LSTM, achieving the largest F1-Score = 75.09% among nonlinear models. This value resulted from a well-balanced hypoglycaemia prediction performance, with a Precision = 79.83% and a Recall = 70.89%. Finally, the low number of false alarms raised by LGB (on average, 1 every 10 days), indicate that this algorithm is a suitable candidate for predicting post-bariatric hypoglycaemic episodes.

### Performance on the test set

Results presented in Table 3 show that, among all the predictive models, rAR achieved the best performance also on the test set by granting F1-score = 73.08%, Precision = 64.38%, Recall = 84.43% with approximately 1 false alarm every 6 days and a median TG of 10 min. Like in the validation set, ARIMA granted an inferior F1-score (about 55%) with respect to rAR due to a drastically deterioration of the Precision (about 45%) and a Recall of about 71%. The FP/day was 0.31 (almost twice the FP/day provided by rAR) and the median time gain was 10 min.

NN provided slightly better performance with respect to ARIMA in terms of F1-score = 59.62% and Precision = 64.14%, but it yielded to a lower Recall = 55.69%, as also reported in the validation set. Compared to rAR, NN raised only 1 false alerts every 10 days with a median time gain of 10 min. Among deep learning models, LSTM achieved the best performance with F1-score = 64.10%, Precision = 68.97% and Recall = 59.88% with 10 min of time anticipation. Compared to rAR, LSTM provide a higher Precision and it granted a lower number of daily false alarms (i.e., less than 1 every 10 days). Finally, CNN-LSTM provided a Precision which is almost equal to the one provided by LSTM (68.70% vs 68.97%, respectively) and an inferior Recall (53.89% vs 59.88%, respectively). The FP/day and the median time gain (i.e., 0.09 and 10 min, respectively) were in line with the other methodologies.

RF provided the highest Precision (70.07%) at the cost of a lower Recall (57.49%) leading to an inferior F1-Score compared to rAR (63.16% vs 73.06%). Additionally, RF raised slightly fewer daily false alarms (0.08 vs 0.17), while the median TG remained similar (10 min). Similarly, LGB provided a Precision = 67.52%, which is comparable with RF (slightly larger than rAR) and a Recall = 63.47% which is instead larger than RF and the other nonlinear models but remained inferior to rAR (84.43%).

An additional assessment of the algorithms, exploiting leave-one-patient-out validation and provided in the Supplementary Material, further confirmed the main findings presented in Table 3.

Table 4 detailed the performance of the CGM-based models in terms of MAE between the CGM traces and predicted profiles for all the PH employed in this work.

**Table 4:** Mean Absolute Error (MAE) on the test set. Results are reported as median [25th-75th] percentile. Abbreviations: FP/day, false positives per day; TG, time gain; rAR, recursive Autoregressive model; ARIMA, Autoregressive Integrated Moving Average; NN, Neural Network; LSTM, Long Short-Term Memory Neural Network; CNN-LSTM, Convolutional Long Short-Term Memory Neural Network; RF, Random Forest; LGB, LightGBM

	**MAE [mg/dL]**			
Model	**PH = 15 min**	**PH = 20 min**	**PH = 25 min**	**PH = 30 min**
rAR	13.18 [11.46–16.10]	18.18 [15.97–22.70]	23.62 [20.87–28.76]	29.77 [24.99–36.82]
ARIMA	11.37 [10.31–13.25]	14.59 [13.00–17.40]	17.54 [15.36–21.05]	19.59 [17.29–23.49]
NN	10.63 [9.70–11.87]	13.63 [12.48–15.02]	16.09 [14.81–18.19]	18.10 [16.36–20.74]
LSTM	10.22 [9.47–11.56]	13.35 [12.35–14.59]	15.89 [14.46–17.99]	18.18 [16.19–20.49]
CNN-LSTM	10.28 [9.49–11.59]	13.35 [12.25–14.68]	15.75 [14.41–17.83]	18.01 [16.07–20.59]
RF	10.85 [9.86–12.39]	13.83 [12.58–16.21]	16.81 [15.59–19.51]	19.16 [17.44–21.87]
LGB	10.90 [9.97–12.46]	14.01 [12.50–16.15]	16.87 [15.41–19.08]	18.88 [17.12–21.55]

Among the models, LSTM is the best performing in terms of predicted glucose profiles, with a MAE spanning from 10.22 mg/dL to 18.18 mg/dL. Other nonlinear models (i.e., NN, CNN-LSTM, RF and LGB) provided similar but slightly larger MAE ranging from about 10.3 to 18.8. The less accurate is rAR with a MAE = 13.18 mg/dL and 29.77 mg/dL, for PH = 15 min and 30 min, respectively. The larger MAE of the rAR model is consistent with findings in the literature [17] and it is attributed to its adaptive nature: to promptly track rapid glucose excursion, rAR is sensitive to noisy data, resulting in an oscillating predicted profile that overestimates high and underestimate low glucose levels.

This could seem inconsistent with the results provided in Table 3, which identifies rAR as the best performing algorithm for PBH forecasting. However, it should be noticed that the MAE only measures the ability of the model to predict glucose levels across the entire glycemic range and it does not account for its performance in predicting hypoglycaemic events that, instead, requires the predicted glucose level to fall below a suitable threshold (i.e., AlarmLevel). Consequently, the underestimation of low glucose levels provided by rAR, although contributing to a higher MAE, is crucial for raising preventive hypoglycemic alarms when current glucose levels are rapidly approaching critically low concentrations.

## Discussion

Working towards a decision support system to help people lowering the burden of post-bariatric hypoglycaemia, we evaluated a set of CGM-based algorithms to forecast hypoglycaemia in real-time. The assessment involved two linear (rAR and ARIMA), three deep learning models (NN, LSTM and CNN-LSTM) and bagging/boosting models (RF and LGB) on the basis of a dataset generated by 47 patients suffering from PBH monitored for about 50 days in unrestricted daily-life conditions.

Among all the models evaluated in this paper, the run-to-run rAR model yielded the best results with PH = 25 min and AlarmLevel = 42 mg/dL, enabling accurate and timely real-time forecasting of PBH events. With a Precision = 64.4%, Recall = 84.4% and 1 false alarm every 6 days, the run-to-run rAR model demonstrated a reasonable balance between sensitivity and number of false alerts. Further, with a Time Gain of 10 min the rAR can provide a sufficient time anticipation for preventive actions (e.g., ingestion of rapid-acting carbohydrates or administration of mini doses of glucagon). The high Recall and the low number of false alarms are important for potential safety and usability claims of hypoglycaemia forecasting algorithms. In fact, missed hypoglycaemia alarms can predispose to patient harm and frequent false alarms can result in unnecessary anxiety and lower responsiveness and disengagement from self-management. Additionally, unnecessary preventive corrections and the related excess of calories due to false alarms predispose to weight gain and rebound hypoglycaemia.

Compared to the other methodologies, rAR achieved the highest F1-score and Recall, while the Precision was only marginally lower than highest observed value (i.e., precision of LSTM was 69%). The slightly inferior Precision might be attributed to the fact that rAR, as a consequence of its adaptivity, is more susceptible to measurement noise, which could trigger false alarms. To compensate for this potential weakness, an optimal tuning of rAR model internal hyperparameters has been performed, which led to set AlarmLevel to 42 mg/dL.

The worst performance in terms of F1-score was achieved by ARIMA and NN (54.88% and 59.62%, respectively). LSTM and CNN-LSTM, which were specifically designed for time series forecasting tasks, both provided a F1-score > 60% with a similar median time gain (10 min) and the lowest number of daily false alarms (i.e., less than 1 every 10 days).

It is noteworthy that unlike under ideal conditions, where one might expect the AlarmLevel to align precisely with the hypoglycaemia threshold (54 mg/dL), the optimal AlarmLevel values selected in the validation set for both rAR and ARIMA models was below this threshold (i.e., 42 mg/dL and 52 mg/dL, respectively). On the contrary, for NN, LSTM, and CNN-LSTM models, the optimal AlarmLevel values were higher, at 59 mg/dL, 57 mg/dL, and 56 mg/dL, respectively.

Our findings suggest that, when using CGM data acquired in unrestricted daily-life conditions, linear models tend to be prone to generate false PBH alerts [31], requiring the threshold to be lowered to compensate for it. On the other hand, the developed deep learning models tend to present a possible positive bias and to overestimate low glucose levels [36], requiring AlarmLevel to be increased to compensate for such a behaviour.

A possible explanation for the positive bias could be linked with the low proportion of glucose values (approximately 1.0%) falling below the hypoglycaemic threshold in the dataset. This may be associated with the behaviour of individuals already diagnosed with PBH, who were trained to take specific measures to prevent hypoglycaemic episodes, such as spacing meals and reducing the carbohydrate intake. Indeed, it is well known from the literature that deep and machine learning algorithms would require balanced datasets [37–39], i.e., with comparable CGM sample size in each glycaemic region, to learn CGM data characteristics appropriately. However, on this dataset, the distribution of CGM samples is highly unbalanced, being ~ 90% in normoglycaemia, ~ 4% in hyperglycaemia, only ~ 5% in hypoglycaemia. This may have played a crucial role in the training process of deep learning models, particularly by leading to the development of predictive models that were more likely to learn the glucose dynamics in the normoglycaemic region where most data samples were concentrated rather than in hypoglycaemia. Similar findings were also reported in [40–42].

Compared to our previous study [12], which identified ARIMA model as the best performing, the current findings show that rAR achieves the best hypoglycaemic prediction performance with the largest F1-Score. This could be attributed to 2 main factors. The first concerns the more challenging real-world dataset employed in this work. In fact, in our previous work, the predictive algorithms were developed and tested in an ideal/noise-free scenario with a limited test set comprising only few patients [8] and few hypoglycemic events (53). Here, the dataset comprises a wide range of individuals with PBH, monitored for 50 days in daily-life conditions, with a total of 167 hypoglycemic events in the test set. The second concerns the adoption of AlarmLevel as a hyperparameter of the hypoglycemia forecasting algorithm. In fact, AlarmLevel acts as a further degree of freedom which allows finding the best trade-off between Precision, Recall and daily false alarms, thus compensating for possibly inaccurate predictions due to the more challenging scenario triggered by the use of free-living condition dataset.

A key aspect of this work concerns the use of real-time CGM data as the only input of the models. As a matter of fact, the developed algorithms can be integrated in a decision support system without requiring any additional intervention from the users. According to [43, 44], the integration of the additional signals generated from wearable devices recorded by IMPACT during the trial, for instance meal information, heart rate or physical activity, into hypoglycaemia prediction algorithms have the potential to improve the predictive performance of glucose dynamics and hypoglycaemia. However, the development of a multi-input predictive algorithm requires a careful balance between the potential benefits, additional technical complexities (e.g., gathering, synchronizing and integrating data from various sources, inaccurate information, interdependencies between signals) as well as user burden (e.g. need to wear additional devices, manual inputs). Further challenges that we experienced in the development of CGM-based PBH prediction algorithms comprised the presence of noise, artefacts, and data gaps due to transmission failures (e.g., due to Bluetooth issues) or temporary sensor errors. A possible solution, partially investigated in [11], is the implementation of real-time filtering techniques as a pre-processing step, prior to model forecasting. However, these techniques introduce an additional delay in the prediction process [16], which can further reduce the time for preventive or corrective actions and thus diminish clinical benefits.

Finally, research progress towards decision support systems for daily management of PBH requires to link hypoglycaemia forecasting algorithms with specific nutritional or drug dosing advice, tailored to individual patient situations and needs [45]. In this context, the metrics of specific predictive algorithm are a critical for the intended use of the decision support. For instance, patients who are regularly engage in high-risk tasks such as driving, and experience frequent and disabling PBH events need a predictive algorithm with high sensitivity able to detect promptly all PBH events (despite the risk of generating some false alarms). Conversely, for people with milder and less frequent episodes a predictive algorithm with high specificity that minimises the nuisance due to false alerts may be preferable. With respect to the development of individualised preventive and/or corrective strategies that are triggered by a predicted event, the implementation of ad-hoc digital twin methodologies has recently emerged as a promising avenue [46–48] but requires further research in the PBH population.

## Conclusions

Real-time CGM-based forecasting of PBH events challenges predictive algorithms due to the rapid post-prandial glucose dynamics, thus allowing little time for generating alerts. Among the different algorithmic approaches evaluated in this study using real world data, a run-to-run forecasting algorithm based on a recursive autoregressive model yielded the most satisfactory balance between recall and false alarms, suggesting that the use of adaptive techniques appears to effectively address real-world glucose dynamics in PBH population. Consequently, future working directions are: i) the development of more complex adaptive models, like regularized latent variables regression methods [49, 50] that allows to exploit exogenous input (e.g., meal intake, exercise information) and to incorporate prior information via suitable kernels (like the stable splines), ii) the development of advanced machine learning models leveraging continual learning in order to update, accumulate and exploit knowledge during the entire training period and iii) the use of learning models founded on accurate and interpretable engineered features (e.g., rate of glucose increase after meal, frequency of hypoglycaemic episodes after meal) rather than relying only on the original CGM history as well as the development of personalized algorithms.

## Supplementary Information

Supplementary Material 1

## Data Availability

The datasets generated and/or analysed during the current study, as well as the code, are not publicly available but are available from the corresponding author on reasonable request.
