# Supplementary material for: Towards a decision support system for post bariatric hypoglycaemia: development of forecasting algorithms in unrestricted daily-life conditions
Source: BMC Med Inform Decis Mak. 2025 Jan 20;25:33. doi: 10.1186/s12911-025-02856-5 (PMC11749296; doi:10.1186/s12911-025-02856-5)
Supplement: Supplementary file 1 — Supplementary Material 1 [file 12911_2025_2856_MOESM1_ESM.docx]

**Supplementary Material**

1. **rAR model: adaptive parameters estimation**

rAR is an adaptive approach employing an autoregressive model based on recursive parameters estimation. Specifically, when a new CGM data is available, a new set of parameters are identified using a recursive least square technique employing the forgetting factor, described as follow:

$$y_{n+1}=\left[ y_{n},y_{n-1},\ldots, y_{n-p+1} \right]\left[ \begin{aligned} a_{1} \\ a_{2} \\ \ldots\\ a_{p} \end{aligned} \right]+v_{n}$$

where the future CGM sample $y_{n+1}$ is described by a linear combination of previous data and model coefficients [$a_{1},\ldots, a_{p}]$, $p$ indicates model order, and additional white noise term $v_{n}$.

In a compact way, it can be rewritten by introducing the term $\Psi_{n+1}^{T}$:

$$y_{n+1}=\Psi_{n+1}^{T}a+v_{n}$$

The weighted recursive least squares can be described as follow:

$$P_{n+1}=\frac{1}{\mu}[P_{n}-\frac{P_{n}\Psi_{n+1}\Psi_{n+1}^{T}P_{n}}{\mu+\Psi_{n+1}^{T}P_{n}\Psi_{n+1}}]$$

$$k_{n+1}= \frac{P_{n}\Psi_{n+1}}{\mu+\Psi_{n+1}^{T}P_{n}\Psi_{n+1}}$$

$$e_{n+1}=y_{n+1}- \Psi_{n+1}^{T}\hat{a}_{n}$$

$$\hat{a}_{n+1}= \hat{a}_{n}+e_{n+1}k_{n+1}$$

where $P$ is the covariance of the data, $k$ can be interpreted as the “gain” that determines how much the prediction error ($e_{n+1}$) affects the update of the parameter estimate ($\hat{a}_{n+1}$). Finally, $\mu$ is the forgetting factor which can be chosen in the range 0-1 and it controls the “memory” of the system. This parameter is usually adopted for modeling non-stationary process and, if properly tuned, it allows to track the rapid changes in glucose levels. For instance, if $\mu$ is low (e.g., 0.2), the model will be very sensitive to rapid changes in data, if $\mu$ is high (e.g., 0.8) the model will provide smoother but more delayed predicted profiles.

1. **Leave-one-patient-out cross validation**

Leave-one-patient-out cross validation (LOPO CV) approach refers to train models on data from all patients except one, and to assess model performance on the left-out patient. This process repeats until each patient has been left out. This strategy allows to better mimic the performance of using a “population-wise” algorithm.

**Table S1** Performance of leave-one-patient-out cross. Results are reported as mean (standard deviation). Abbreviations: FP/day, false positives per day; TG, time gain; rAR, recursive Autoregressive model; ARIMA, Autoregressive Integrated Moving Average; NN, Neural Network; LSTM, Long Short-Term Memory Neural Network; CNN-LSTM, Convolutional Long Short-Term Memory Neural Network; RF, Random Forest; LGB, LightGBM.

| **Model** | **Precision (%)** | **Recall (%)** | **FP/day** | **TG (min)** |
| --- | --- | --- | --- | --- |
| **rAR** | 67.62 (23.05) | 84.43 (15.09) | 0.12 (0.2) | 10 (7) |
| **ARIMA** | 42.86 (22.50) | 67.13 (20.43) | 0.27 (0.2) | 10 (5.7) |
| **NN** | 63.35 (24.10) | 52.27 (24.15) | 0.1 (0.2) | 10 (5.3) |
| **LSTM** | 67.25 (22.44) | 57.95 (22.01) | 0.07 (0.1) | 10 (3.9) |
| **CNN-LSTM** | 63.98 (24.17) | 55.16 (21.57) | 0.08 (0.1) | 10 (2.9) |
| **RF** | 70.44 (22.50) | 54.59 (24.34) | 0.07 (0.06) | 10 (2.9) |
| **LGB** | 67.54 (21.69) | 63.21 (23.11) | 0.1 (0.1) | 10 (3.2) |

Table S1 shows the hypoglycaemia prediction metrics in terms of mean and standard deviation over the 47 runs of the LOPO-CV. The best performing algorithm is rAR which provides the highest Recall (84.43%) and Precision (67.62%) with only few false alarms per day and a Time Gain which is 10 minutes on average. ARIMA provides the lowest Precision (42.86%) and the largest FP/day. NN, LSTM and CNN-LSTM grant a Precision ranging from 63.35% to 67.25% but a low Recall varying from 52.27% to 57.95%. Compared to the neural network models, RF and LGB provide larger Precision (70.44% and 67.54%, respectively) and comparable, yet slightly larger, Recall (54.59% and 63.21%, respectively).

A note of caution is necessary when interpreting the large standard deviation (ranging from 15.09% to 24.34%) for Precision and Recall of all the algorithms. As a matter of fact, leveraging single patient data as test set (as required by the LOPO-CV) presents only a limited number of hypoglycaemic episodes (on average, 4 every 10 days for subject). As a consequence, this single-patient level analysis can be strongly quantized: for instance, in a patient with 2 hypoglycemic episodes, recall can take only three values: 100%, 50% or 0%.
